# Supplementary material for: Spatiotemporal recruitment of RhoGTPase protein GRAF inhibits actomyosin ring constriction in Drosophila cellularization
Source: eLife. 2021 Apr 9;10:e63535. doi: 10.7554/eLife.63535 (PMC8081525; doi:10.7554/eLife.63535)
Supplement: Supplementary file 1. — The stocks and recombinants used in this study are numbered in Table 1. The crosses carried out with the stocks for each figure for live imaging and fixed imaging of embryos along with the temperature at which each cross is carried out are detailed in Table 2. [file elife-63535-supp1.docx]

**Supplemental file 1: *Drosophila* recombinants and crosses**

The *Drosophila* stocks are labelled numerically in table 1. The stock numbers in rows 20-29 were made with standard genetic crosses. The F2 embryos for live imaging or fixed staining were obtained from crosses shown in Table 2 at the given temperature.

**Table 1: *Drosophila* stocks and recombinants**

| **Stock** | **Genotype** | **Source/Reference** |
| --- | --- | --- |
| 1 | Canton-S | Lab stock originally obtained Bloomington *Drosophila* Stock Center, BDSC #1 |
| 2 | *nanos*-Gal4 | Lab stock ([Mavrakis et al., 2009, 2008)](https://paperpile.com/c/YxeQem/W0z6Y+fAXN) |
| 3 | w; *mat67*-Gal4; *mat15*-Gal4 | Girish Ratnaparkhi, IISER, Pune, India |
| 4 | w; P{Sqh-mCherry.M}3 | Bloomington *Drosophila* Stock Center (BDSC), Indiana, USA |
| 5 | y[1] v[1]; P{TRiP.HMC03427}attP40 (GRAF shRNA1) | BDSC, #51853 |
| 6 | y[1] v[1]; P{TRiP.GL01207}attP40 (myosin binding subunit, MBS shRNA) | BDSC, #41625 |
| 7 | y[1] sc[*] v[1] sev[21]; P{TRiP.HMS01118}attP2 (RhoGEF2 shRNA) | BDSC, #34643 |
| 8 | y[1] w[*]; P{UASpT7.RhoGEF2}5 (RhoGEF2 overexpression) | BDSC, #9386 |
| 9 | *ubi-*GFP::AnillinRBD/TM3 | Munjal et al., 2015, Thomas Lecuit, France |
| 10 | w^-^FM7a/w^-^FM7a(white eye) | Girish Ratnaparkhi, IISER, Pune, India |
| 11 | UASp-rok-shRNA (*rok*^i^) | Zhang et al., 2018 |
| 12 | y[1] w[67]c[23] P{EPgy2}Graf[EY09461] (GRAF overexpression) | BDSC, #17571 |
| 13 | w;*mat67* Spider-GFP-Sqh-mcherry/TM3ser | Martin et al., 2009 |
| 14 | y[1] sc[*] v[1] Graf[CR57]/FM7a (*Graf*^CR57^) | Crispr mutant of *Graf* generated in this study |
| 15 | y[1] sc[*] v[1] sev[21]; P{TKO.GS00762}attP40 | BDSC, #76993 |
| 16 | P{KK102763}VIE-260B (GRAF shRNA2, *Graf*^2i^) | Vienna *Drosophila* Stock Center, #v110812 |
| 17 | y[1] sc[*] v[1] sev[21]; P{y[+t7.7] v[+t1.8]=nos-Cas9.R}attP40 | BDSC #78781 |
| 18 | [w]*;p[UASp-GRAF-EGFPG1] attp40/cyo | Full length GRAF-GFP generated in this study |
| 19 | [w]*;p[UASp-GRAFΔRhoGAP-GFP] attp40/cyo | GRAF lacking the RhoGAP domain generated in this study |
| 20 | y[1] w[67]c[23] P{EPgy2}Graf[EY09461]/FM7a; P{UASpT7.RhoGEF2}5/Tb (GRAF-OE;RhoGEF2-OE) | Recombinant containing overexpression of GRAF and RhoGEF2 generated for this study |
| 21 | y[1] sc[*] v[1] sev[21]; P{TRiP.HMC03427}attP40/cyo;P{TRiP.HMS01118}attP2/Tb (*Graf*^i^;*RhoGEF2*^i^) | Recombinant containing GRAF RNAi1 and RhoGEF2 RNAi generated in this study |
| 22 | mat67-Sqh-mCherry; *ubi-*AnillinRBD-GFP-NG4/Tb | Recombinant containing mat67-Gal4, Sqh-mCherry and *ubi*-AnillinRBD-GFP generated for this study |
| 23 | y[1] sc[*] v[1] GRAF[CR57]/FM7a; P{TRiP.HMS01118}attP2/Tb (*Graf*^CR57^;*RhoGEF2*^i^) | Recombinant containing the Crispr mutant of *Graf* and RhoGEF2 RNAi generated for this study |
| 24 | y[1] sc[*] v[1] GRAF[CR57]/FM7a; UASp-rok-shRNA/Tb(*Graf*^CR57^;rok^i^) | Recombinant between the Crispr mutant of *Graf* and Rok RNAi generated for this study |
| 25 | y[1] w[67]c[23] P{EPgy2}Graf[EY09461]/FM7a; P{TRiP.GL01207}attP40/cyo (GRAF-OE;*mbs*^i^) | Recombinant between overexpression of GRAF and RNAi for *mbs* generated for this study |
| 26 | y[1] sc[*] v[1] GRAF[CR57]/FM7a;mat67, Sqh-mCherry/CyO | Recombinant between the Crispr mutant of *Graf*, mat67-Gal4 and Sqh-mCherry generated for this study |
| 27 | y[1] sc[*] v[1] GRAF[CR57]/FM7a; p[pUASp GRAF EGFP G1] attp40/cyo | Recombinant between Crispr mutant of *Graf* and GRAF-GFP generated for this study |
| 28 | y[1] sc[*] v[1] GRAF[CR57]/FM7a; p[pUASp GRAF RhoGAP del EGFP] attp40/cyo | Recombinant between Crispr mutant of *Graf* and GRAF lacking the RhoGAP domain with GFP tag generated for this study |
| 29 | y[1] sc[*] v[1] GRAF[CR57]/FM7a; *ubi*-AnillinRBD-GFP/cyo | Recombinant between the Crispr mutant of *Graf* and *ubi*-AnillinRBD-GFP generated for this study |

**Table 2: *Drosophila* crosses**

| **F2 embryos were imaged from these crosses, using above stock numbers/genotypes. Non-balancer females were used for cages.**  Stocks # (Virgins x males) | **Figure** | **Temperature (^0^C)** |
| --- | --- | --- |
| 2 x 1 control | 1C,D and 1-figure supplement 2A | 28 |
| 2 x 5 *Graf*^i^ using *nanos*-Gal4 | 1C,D and 8B | 28 |
| 3 x 16 *Graf*^2i^ using *mat*-Gal4 | 1-figure supplement 2A | 28 |
| 17X15 *Gra*f^gRNA^ using *nanos*-Cas9 | 1-figure supplement 2A | 28 |
| 14x14 *Graf*^CR57^ | 1C,D; 2A; 6A | 25 |
| 26X27 *Graf*^CR57^;GRAF-GFP/*mat*67-Gal4, Sqh-mCherry | 2D; 4F; 4-figure supplement 1A | 18 |
| 22X1 mat67 Sqh-mCherry/+; *ubi*-AnillinRBD-GFP NG4/+ | 4A | 25 |
| 26X29 *Graf*^CR57^;*ubi*-AnillinRBD-GFP /*mat*67-Gal4, Sqh-mCherry | 4C | 25 |
| 4X4 Sqh-mCherry | 3A,E | 25 |
| 13x1 *mat67-*Gal4 Spider-GFP-Sqh-mcherry/+ | 4F:5D; 6C; 7A; 8A; 9A | 25 |
| 26X26 *Graf*^CR57^;*mat67*-Gal4, Sqh-mCherry | 3A,E; 4F; 6C; 8A; 9A; 6-figure supplement 1A,B | 25 |
| 26x28 *Graf*^CR57^;GRAFΔRhoGAP-GFP/*mat67*-Gal4, Sqh- mCherry | 4F; 4-figure supplement 1A,B | 18 |
| 3x12 GRAF-OE using *mat*-Gal4 | 5B; 7B | 28 |
| 13X12 GRAF-OE; *mat67* Spider-GFP-Sqh-mCherry | 5D; 6C; 7A; 6-figure supplement 1A,B | 25 |
| 3X8 RhoGEF2-OE using *mat*-Gal4 | 7,B; 7-figure supplement 1A | 25 |
| 13X8 RhoGEF2-OE;*mat67* Spider-GFP-Sqh-mCherry | 7A; 7-figure supplement 1B | 25 |
| 2X7 *RhoGEF2*^i^ using *nanos*-Gal4 | 8B | 28 |
| 3X7 *RhoGEF2*^i^ using *mat*-Gal4 | 8-figure supplement 1 | 25 |
| 13X7 *RhoGEF2*^i^ ;*mat67*-Gal4 Spider-GFP-Sqh-mCherry | 8A; 7-figure supplement 1B | 25 |
| 13X20 GRAF-OE;RhoGEF2-OE/*mat67*-Gal4 Spider-GFP, Sqh-mCherry | 7A | 25 |
| 3X20 GRAF-OE;RhoGEF2-OE using *mat*-Gal4 | 7B | 28 |
| 2X21 *Graf*^i^;*RhoGEF2*^i^ using *nanos*-Gal4 | 8B | 28 |
| 3X6 *mbs*^i^ using *mat*-Gal4 | 9-figure supplement 1 | 25 |
| 3X25 GRAF-OE;*mbs*^i^ using *mat*-Gal4 | 9-figure supplement 1 | 25 |
| 13x11 *rok*^i^/*mat67*-Gal4 Spider-GFP-Sqh-mCherry | 9A | 25 |
| 13X24 *Graf*^CR57^;*rok*^i^/*mat67-*Gal4 Spider-GFP-Sqh-mCherry | 9A | 25 |
